# Tracking tumor alteration in glioma through serum fibroblast activation protein combined with image

**DOI:** 10.1186/s12885-023-11544-4

**Published:** 2023-10-20

**Authors:** Xiao-song Yang, Peng zhu, Rong-Xing Xie, Peng-fei Chen, Hong Liu, Xiao-Man Cheng, Zheng-Quan Zhu, Xiao-min Peng, Hai-bin Liu, Qun-Ying Yang, Jun-Qi Li, Ji Zhang

**Affiliations:** 1 Department of Neurosurgery, State Key Laboratory of Oncology in South China, Collaborative Innovation Center for Cancer Medicine, Sun Yat-sen University Cancer Center, Guangzhou, China; 2 Department of Anesthesiology, State Key Laboratory of Oncology in South China, Collaborative Innovation Center for Cancer Medicine, Sun Yat-sen University Cancer Center, Guangzhou, China; 3 Department of Laboratory Medicine, State Key Laboratory of Oncology in South China, Collaborative Innovation Center for Cancer Medicine, Sun Yat-sen University Cancer Center, Guangzhou, China; 4 Department of neurosurgery, Tumor Hospital Affiliated of Xinjiang Medical University, Xinjiang, China; 5 State Key Laboratory of Respiratory Diseases, National Clinical Research Center for Respiratory Diseases, Guangzhou Institute of Respiratory Health, First Affiliated Hospital of Guangzhou Medical University, Guangzhou, China; 6 Department of Imaging Diagnosis Center, State Key Laboratory of Oncology in South China, Collaborative Innovation Center for Cancer Medicine, Sun Yat Sen University Cancer Center, Guangzhou, China; 7 Third Affiliated Hospital of Zhengzhou University, Zhengzhou, China

**Keywords:** Glioma, Fibroblast activation protein, Image, Serum, Dynamic detection

## Abstract

**Purpose:**

Detecting tumor progression of glioma continues to pose a formidable challenge. The role of fibroblast activation protein (FAP) in gliomas has been demonstrated to facilitate tumor progression. Glioma-circulating biomarkers have not yet been used in clinical practice. This study seeks to evaluate the feasibility of glioma detection through the utilization of a serum FAP marker.

**Methods:**

We adopted enzyme-linked immunosorbent assay (ELISA) technique to quantify the relative FAP level of serum autoantibodies in a cohort of 87 gliomas. The correlation between preoperative serum autoantibody relative FAP levels and postoperative pathology, including molecular pathology was investigated. A series of FAP tests were conducted on 33 cases of malignant gliomas in order to ascertain their efficacy in monitoring the progression of the disease in relation to imaging observations. To validate the presence of FAP expression in tumors, immunohistochemistry was conducted on four gliomas employing a FAP-specific antibody. Additionally, the investigation encompassed the correlation between postoperative tumor burden, as assessed through volumetric analysis, and the relative FAP level of serum autoantibodies.

**Results:**

A considerable proportion of gliomas exhibited a significantly increased level of serum autoantibody relative FAP level. This elevation was closely associated with both histopathology and molecular pathology, and demonstrated longitudinal fluctuations and variations corresponding to the progression of the disease The correlation between the rise in serum autoantibody relative FAP level and tumor progression and/or exacerbation of symptoms was observed.

**Conclusions:**

The measurement of serum autoantibody relative FAP level can be used to detect the disease as a valuable biomarker. The combined utilization of its detection alongside MR imaging has the potential to facilitate a more accurate and prompt diagnosis.

## Introduction

Glioma as the most common primary malignant brain tumor in adults, is regarded as one of the leading causes of cancer death worldwide [1, 2]. Despite notable advances in therapy, patients with glioma, particularly those with high-grade glioma, persistently experience an unfavorable prognosis. According to a multicentric data, the median overall survival of glioblastoma (GBM) patients is approximately 15 months, with a 5-year survival rate of < 10% [1, 3].

The resected tumor or biopsy tissue allows direct access to genetic information or immunohistochemical biomarkers in glioma. Multiple molecular biomarkers have been identified from the tumor, including isocitrate dehydrogenase 1 and 2 (IDH1/2), codeletion of chromosome arms 1p and 19q (1p/19q codeletion), and O-6-methylguanine-DNA methyltransferase (MGMT), which play important roles in patient stratification, delineation of risk groups, and prognostication of treatment response, among other aspects [4]. Tissue specimens acquired via a highly invasive procedure present substantial clinical risk. Furthermore, the implementation of repeated tumor tissue sampling in clinical practice is deemed entirely impractical. The assessment following treatment is currently predicated exclusively on the consecutive analysis of magnetic resonance imaging (MRI), which is assessed using the modified RANO criteria [5, 6]. Therefore, in contrast to other types of tumors, the incorporation of verified circulating biomarkers into the diagnosis and treatment of glioma has not yet been achieved. MRI serves as a conventional modality for glioma imaging and demonstrates effectiveness; however, it possesses the potential to yield misleading results due to hysteresis and pseudoprogression. The monitoring of early-stage glioma relapse through exclusive reliance on MRI-based detection is challenging. Therefore, there is an urgent need to develop a widely accessible and minimally invasive method for tracking glioma. The monitoring of glioma progression should incorporate the utilization of tumor-based circulating biomarkers as an adjunctive parameter. In certain circumstances, when the likelihood of tumor recurrence is uncertain, the inclusion of supplementary detection would be highly advantageous in facilitating clinical decision-making.

FAP is a membrane protease in cancer-associated stromal fibroblasts and contributes to tumor progression but is absent or insignificant in most normal tissues [7–9]. The findings from immunohistochemical analyses conducted on extracranial tumor tissues indicate that elevated FAP expression is indicative of an unfavorable prognosis, suggesting a significant involvement of FAP in tumorigenesis [10]. Histopathological studies revealed that FAP expression was elevated in gliomas, particularly in mesenchymal subtypes [11]. Although FAP has been extensively investigated as a biomarker in various cancer types, there is currently a lack of studies reporting on the longitudinal monitoring of glioma progression using sequential serum FAP.

In the present study, we conducted an investigation to identify the presence of the serum marker FAP and assess its viability as a means of monitoring the progression of glioma. Based on our findings, it can be inferred that the integration of serum autoantibody relative FAP level and MRI examination has the potential to enhance the precision of tumor progression monitoring in a clinical setting.

## Materials and methods

### Patients

From February 2020 to May 2021, 87 glioma patients (47 males and 40 females, median age 48.2 years, range 18–74 years) were recruited for this study at the Affiliated Tumor Hospital of Xinjiang Medical University, the Third Affiliated Hospital of Zhengzhou University and the Sun Yat-Sen University Cancer Center. These patients met the following inclusion and exclusion criteria: no combined tumors other than gliomas, age > 18 years, no cancer history, and no prior anti-cancer therapy before blood collection. Following tumor removal, all patients received standard care. Glioma was classified using the current World Health Organization (WHO) Classification Criteria. All patients were diagnosed using pathological examination and histological specimens were confirmed by two independent experienced pathologists. Clinical characteristics were collected from the medical records. All research methods and experiments were conducted under the applicable regulations. The study was authorized by the medical ethics committee (SL-B2022-567-01). Individual participants or their family members provided written informed consent.

### Blood sample collection

Blood samples were obtained from 87 patients diagnosed with glioma through the collection of cubital vein specimens, which were subsequently incubated in 3 ml tubes containing ethylene-diamine tetra acetic acid dipotassium salt. Among them, 33 patients diagnosed with high-grade gliomas were enrolled in the study, with the objective of obtaining a minimum of two consecutive peripheral venous blood samples during the course of adjuvant chemotherapy. All these samples were rested for 30 min at room temperature before being centrifuged at 3500 rpm at 4℃ for 10 min to separate the serum. The supernatants were collected and aliquoted into 250 µl cryo-tubes, which were then stored in liquid nitrogen until the ELISA technique was employed to ascertain the relative level of the FAP antigen.

### ELISA for blood samples

The plates were coated with 100 ng of recombinant FAP extracellular protein per well and incubated overnight at 4℃. After blocking the plates for 2 h at room temperature with 300 µl blocking buffer (10 mM pH 7.4 PBS with 15% goat serum), the serum specimens were added to the wells at 37℃ for 1 h and washed 5 times. Another 1 h at 37 °C was spent incubating 100 µl of diluted HRP-labeled goat anti-human IgG antibody (Promega, USA). The plates were washed 3 times with the washing buffer before being treated with 100 µl TBM per well for 15 min at 37℃ and 50 µl 2 M H_2_SO_4_ to block the response. The optical density of each well within 5 min was determined using a microplate reader set to 450 nm.

### Immunohistochemistry (IHC) on human glioma tissue

IHC was conducted according to the manufacturer’s instructions to detect FAP expression in glioma tissue. Briefly, following the segmentation of formalin-fixed paraffin-embedded (FFPE) tissue specimens with a thickness of 0.4 μm, the slides underwent a deparaffinization and rehydration process utilizing progressive ethanol gradients. High-temperature antigen extraction was conducted in 10 mM/ml citrate buffer (pH 6.0) for 30 min. Endogenous peroxidase was quenched twice for 15 min with 3% hydrogen peroxide. Primary FAP-specific antibody (Promega, USA) was incubated for FAP staining in glioma slides. Finally, two neuropathologists conducted counterstaining, observation, and photography of the slides.

### MR image examination

All patients in the study underwent preoperative and postoperative MRI scans on a regular basis. Following the completion of each postoperative FAP test, a corresponding multimodal MRI examination was accessible. The same multiplex scanner model, the protocol for acquisition, and reconstruction software were used for these patients. All MRI examinations were conducted using contrast agents and subsequently analyzed in a retrospective manner. The radiologists were aware of the pathological diagnosis of gliomas for the tumors, yet they did not know knowledge regarding the treatment details of the patients. Two imaging specialists, each possessing over a decade of experience, conducted an autonomous analysis. The resolution of disputed results was achieved through expert discussion in accordance with pertinent guidelines and consensuses. Tumor volumes were automatically calculated using computer-aided planimetric software.

### Data analyses

The statistical analyses were conducted utilizing R (version 4.3.1). The Shapiro-Wilk test was employed to assess normality, while Bartlett’s statistic was utilized to evaluate homogeneity of variances. If the data met the criteria of normal distribution and homogeneity of variance, the statistical tests employed were Student’s t-test for two groups or ANOVA for more than two groups. In instances where the data did not exhibit conformity to a normal distribution and homogeneity of variance, the Mann-Whitney U test was employed for two groups, while the Kruskal-Wallis test was utilized for more than two groups, in order to conduct data analysis (*p < 0.05, **p < 0.01, ***p < 0.001).

## Results

### Serum FAP level is elevated in the majority of human gliomas

**Fig. 1:**
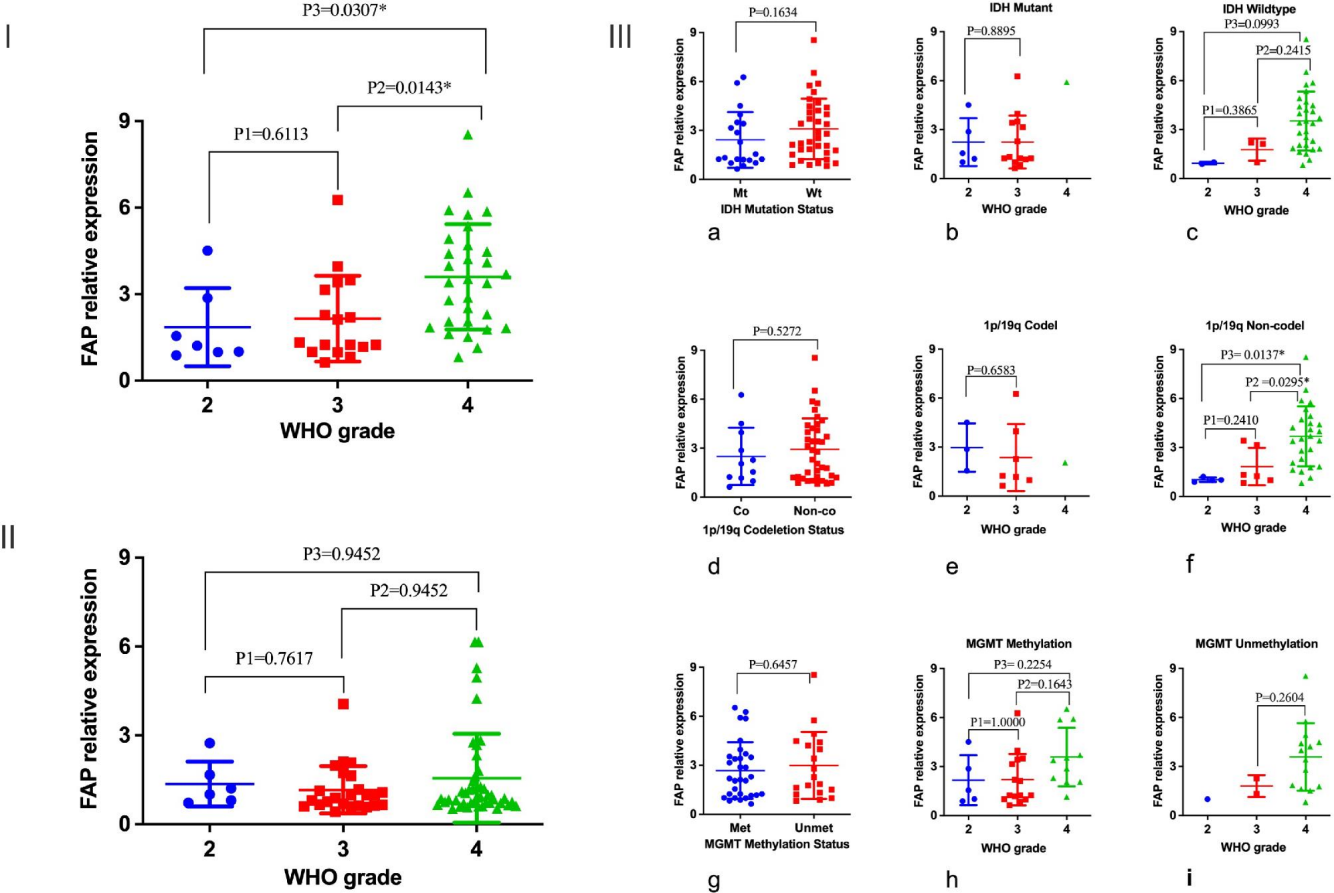
The relative level of preoperative serum FAP autoantibodies in a cohort of 87 patients with different WHO-grade gliomas was assessed using the enzyme-linked immunosorbent assay (ELISA) (**I**). The scatterplot depicts the autoantibody relative serum FAP level in postoperative 87 patients with grade 2–4 glioma (**II**). The correlation between serum autoantibody relative FAP level and molecular pathology in gliomas has been established. The levels of serum autoantibodies relative to FAP in IDH-mutant gliomas are found to be significantly elevated compared to IDH-wild type gliomas of the same grades 2 and 3. FAP expression is more common in IDH-wildtype GBMs than in IDH-mutant gliomas with WHO 4 grade (**III a, b, c**). Serum autoantibody relative FAP levels are higher in 1p/19q codeleted gliomas than in 1p/19q non-codeleted gliomas of identical grades 2 and 3. The likelihood of FAP level is higher in 1p/19q non-codeleted glioblastomas (GBMs) compared to 1p/19q codeleted gliomas with WHO 4 grade (**III d, e, f**). Serum autoantibody relative FAP levels The levels of serum autoantibodies relative FAP are typically elevated in MGMT-methylated gliomas compared to MGMT-unmethylated gliomas. There is no apparent distinction observed between GBMs with MGMT methylation and those without methylation (**III g, h, i**)

The scatter plot depicted the preoperative and postoperative levels of serum autoantibody relative FAP in the 87 glioma patients, demonstrating that the serum FAP levels were distinct in various histopathological grades (Fig. 1I-II). The findings revealed a notable elevation in serum FAP levels among the majority of high-grade gliomas. Serum levels of FAP were compared in relation to pathological findings and molecular pathology. There was a notable rise in FAP levels observed as the tumor grade increased, as shown in Fig. 1I. The levels of serum autoantibody relative to FAP generally exhibited a decrease three days following surgery in comparison to the preoperative levels (Fig. 1 II). Distinct characteristics of serum FAP levels can be observed in various grades of gliomas, as determined by the molecular phenotype (Fig. 1 III). In terms of IDH mutation status, FAP level was slightly lower in patients with IDH-mutant glioma compared to the IDH-wildtype, but the difference was not statistically significant (Fig. 1IIIa, P = 0.1634). There was no statistically significant disparity observed in FAP levels between grade 2 and grade 3 gliomas within the IDH-mutant glioma cohort(Fig. 1IIIb; P = 0.8895), whereas in the cohort of IDH-wildtype glioma patients, a elevation in serum FAP levels was observed specifically among grade 4 gliomas, but this differecne did not reach statistical significance (Fig. 1 IIIc; P1 = 0.3865, P2 = 0.2415, P3 = 0.0993). The levels of serum FAP were found to be lower in glioma patients exhibiting 1p/19q codeletion compared to those with 1p/19q non-deletion (Fig. 1IIId; P = 0.5272). In relation to the 1p/19q codeletion, the serum FAP level was found to be higher in grade 2 glioma compared to grade 3 glioma (Fig. 1IIIe; P = 0.6583). This difference was particularly evident among patients diagnosed with grade 4 glioma in comparison to those with grade 2 and grade 3 glioma. (Fig. 1 IIIf; P2 = 0.0295, P3 = 0.0137). Irrespective of the methylation status of the MGMT promoter, elevated levels of FAP were observed in gliomas of all grades, regardless of whether they were methylated or unmethylated. There is no statistically significant disparity in serum FAP levels observed between patients exhibiting methylated and unmethylated conditions (Fig. 1 IIIg; P = 0.6457). In relation to MGMT methylation, serum FAP levels show no discernible differences between grade 4 glioma and grade 2 glioma, and grade 3 glioma (Fig. 1 IIIh; P2 = 0.1643, P3 = 0.2254). The serum FAP level was found to be higher in grade 4 glioma compared to grade 3 glioma among patients with MGMT unmethylation (Fig. 1 IIIi; P = 0.2604). However, there was significant FAP expression heterogeneity among identical graded gliomas (Fig. 1; Table 1).

**Table 1:** Clinical and pathological characteristics of four gliomas

Patients	Age (years)	Gender	Diagnosis	Localization	Serum FAP	IDH mutation (Y/N)	1p\19q co-del (Y/N)	MGMTpm status
1	62	M	DA	RTL	0.99	N	Y	N
2	61	M	GBM	LTL	5.75	N	N	N
3	33	F	AA	RTIL	1.33	Y	N	N
4	34	F	AO	LFL	2.87	Y	Y	Y

*DA* diffuse astroglioma, *AA* anaplastic astrocytoma, *AO* anaplastic oligodendroglioma, *RTL* right temporal lobe, *LTL* left temporal lobe, *RTIL* right temporal-insular lobe, *LFL* left frontal lobe, Y means IDH1 R132H Mutation, N means Wildtype, *PM* promoter methylation

### FAP expression in glioma tissues

**Fig. 2:**
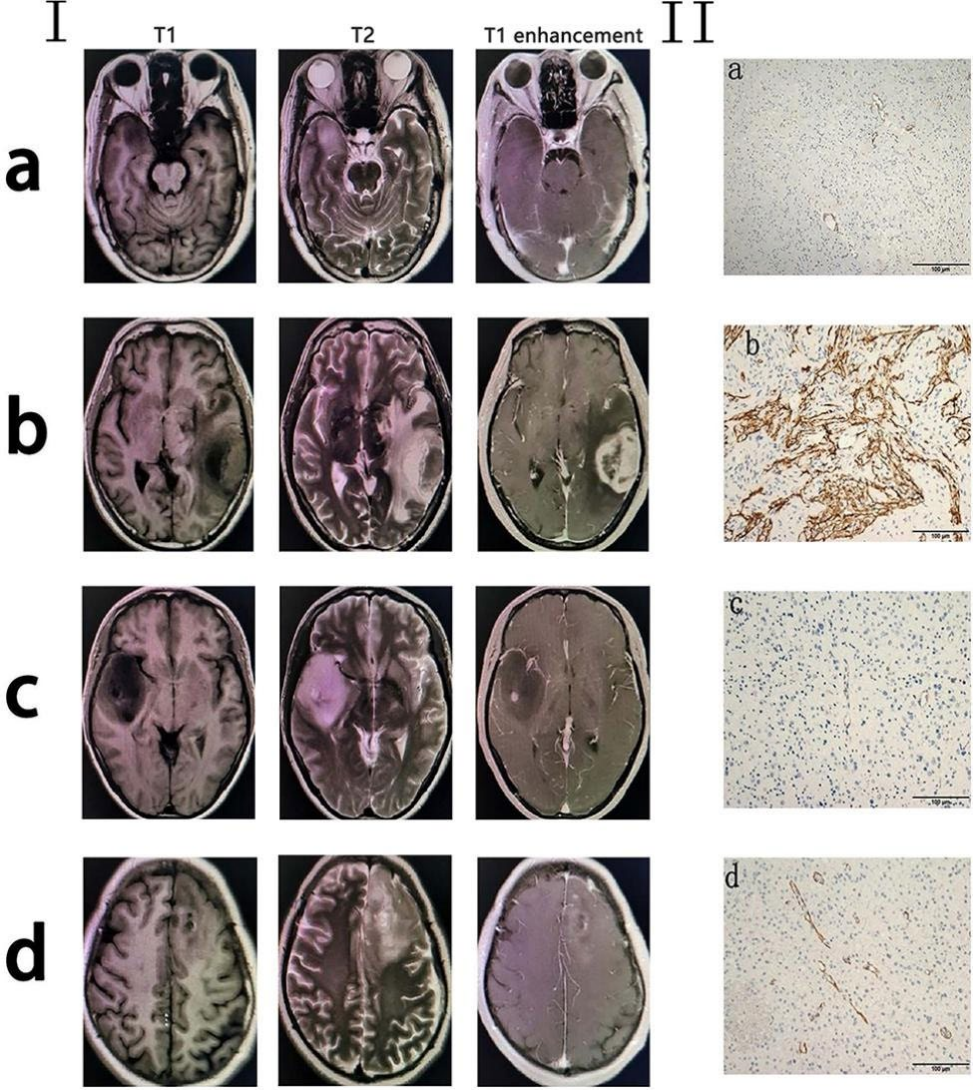
All the images exhibit axial orientation, displaying T1 and T2-weighted intensity, as well as T1-weighted intensity following contrasted enhancement. The presented case exhibits the presence of a mass in the medial temporal lobe on the right side, characterized by an indeterminate border and lack of enhancement (**I a**).The presented case exhibits the presence of left temporal lobe glioblastoma, characterized by heterogeneous enhancement and occupying effects (**I b**). The present case involves an anaplastic astrocytoma (WHO grade 3) located in the right temporal insular lobe. Notably, there is evident peripheral edema surrounding the tumor, along with a central dot-shaped enhancement (**I c**). The case unveiled the presence of a mass in the left frontal lobe, accompanied by edema and sporadic enhancement (**I d**). Immunohistochemistry affirms the wide expression of FAP in glioma tissue. Typical immunohistochemistry image of FAP expression was represented in the four glioma patients’ section. Tumor tissue sections were stained to determine FAP expression (brown). In many areas, spindle cells are strongly stained for FAP, which was also observed in cells surrounding blood vessels (**II a-d**). FAP-positive cells proliferate primarily around microvascular protrusions in GBM (**II b**). Scale bars 100 μm in a for a–d (**II**)

Four glioma patients’ paraffin-embedded tissues were available (Fig. 2 I). The 4 FFPE specimens were histologically verified as gliomas (Table 1). The degree of FAP staining observed in the primary tumor parenchyma was found to be directly proportional to the concentration of serum FAP in these individuals. Figure 2 II illustrates four specific examples of IHC staining, demonstrating that the expression of FAP is consistently confined to the primary tumor tissue. FAP expression was found in these tumors in two distinct patterns, as shown in Fig. 2 II. All four specimens exhibited varying degrees of FAP-positive fields, with a predominant localization of strong FAP staining observed in the fibrotic stromal bands across the majority of samples (Fig. 2 II). Additionally, a disparity in FAP expression was observed between GBM and grade 2 and 3 glioma. In IDH-Wildtype diffuse astroglioma, FAP-positive cells were extremely scarce.

### Blood FAP level and its relation to MR image

**Fig. 3:**
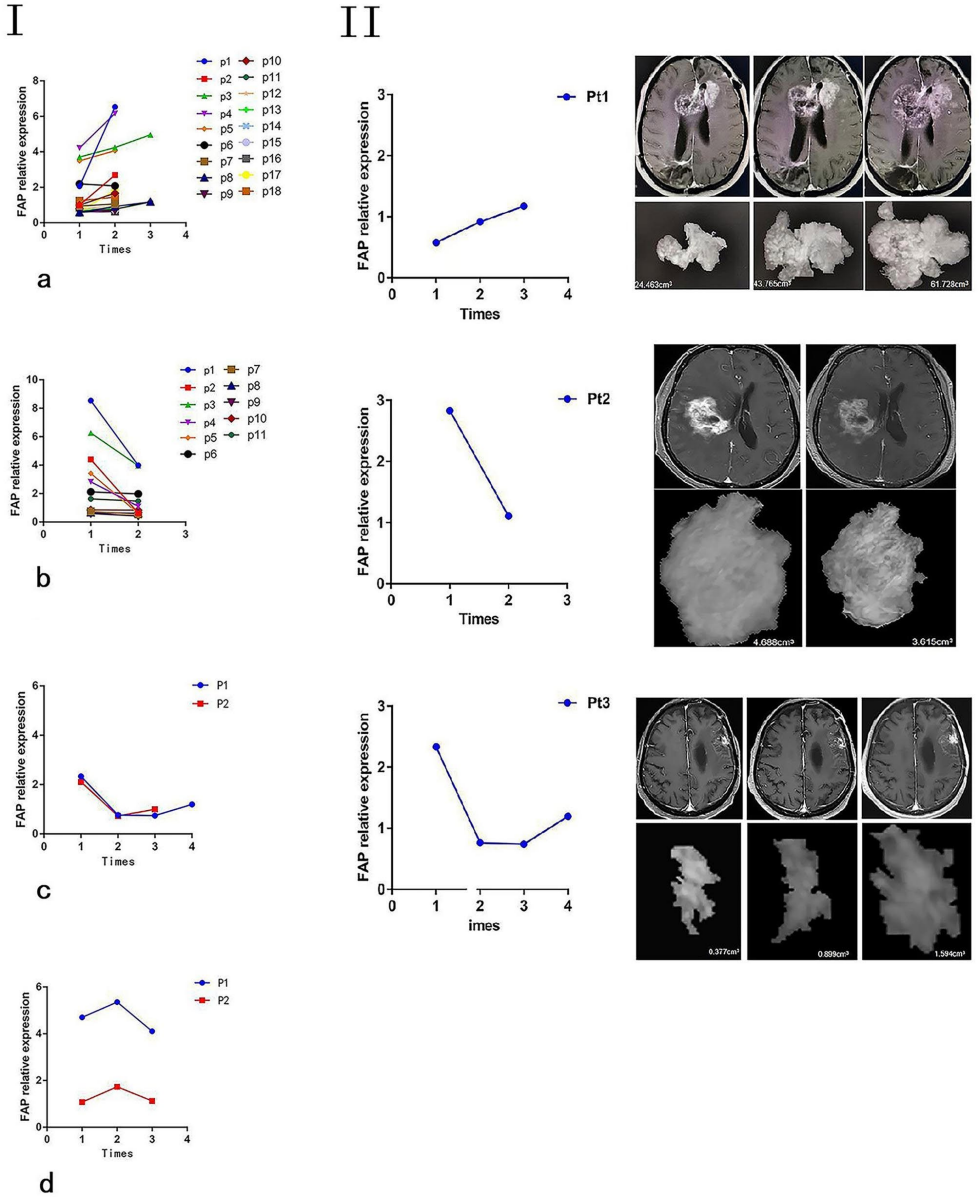
Longitudinal bead-assisted ELISA data from 33 glioma patients show dynamic variation in serum autoantibody relative FAP levels, as well as tumor status. The levels of serum autoantibody relative FAP exhibite a significant increase during the progression of the tumor, followed by a substantial decrease upon disease remission. There was no significant variation observed in FAP levels among patients with stable tumors (**I**). During adjuvant chemotherapy, a positive correlation was observed between serum autoantibody relative FAP levels and tumor volume (**II**)

On the same day, a craniocerebral MRI scan and a blood draw for the familial adenomatous polyposis FAP test were conducted. It is noteworthy to mention that each of the cases exhibited FAP expressions. Furthermore, the blood specimens from the 33 patients were analyzed in matched pairs to assess the serial detection of serum FAP. The findings revealed a positive correlation between the level of FAP and tumor volume as observed on MR images. In order to ascertain the potential correlation between changes in serum FAP levels and the progression of glioma during treatment, a series of serum samples were collected from a cohort of 33 patients for the purpose of FAP analysis (Fig. 3 I). The study observed an increase in serum FAP levels as tumor progression, while a decrease in these levels was observed following chemotherapy. In instances where the tumor remains stable, there is an absence of notable variations in serum FAP levels. In the 33 cases, a minimal or imperceptible lesion was observed in the surgical area. A substantial correlation was observed between the level of serum autoantibody relative FAP and tumor volume among the subjects (Table 2). Three patients, selected as representative cases, were chosen to exemplify varying degrees of tumor progression or elimination. Their intracranial lesions were identified using an MRI scan and tumor burden was calculated using computer-aided planimetric analysis. Most importantly, the levels of serum FAP exhibited a decline in response to therapy, while conversely, they demonstrated an increase in the presence of tumor progression (Fig. 3 II). In cases where there is incongruity between the MRI findings and clinical symptoms, the inclusion of a blood FAP test can serve as an indirect means of confirming the presence of tumor progression that may not be visually detectable. During the post-treatment follow-up, it was observed that the serum FAP level was elevated in cases where clinical manifestations of tumor progression were present, as opposed to those without any clinical symptoms. However, there was no significant difference observed in the results of imaging examinations.

**Table 2:** The correlation between serum FAP level and tumor volume during adjuvant chemotherapy

Patients	Age (years)	Gender	Diagnosis	FAP_1_ / TV_1_(cm^3^)	FAP_2_ / TV_2_(cm^3^)	FAP_3_ / TV_3_(cm^3^)	FAP_4_ / TV_4_(cm^3^)
1	39	F	GBM, IDH(-)	0.58 / 24.46	0.92 / 43.77	1.17 / 61.73	- / -
2	57	F	GBM, IDH(-)	2.83 / 4.69	1.11 / 3.62	- / -	- / -
3	72	F	GBM, IDH(-)	2.33 / 1.59	0.76 / -	0.74 / 0.38	1.2 / 0.9

*TV* tumor volume

## Discussion

Precise diagnosis, to an extreme, can improve the efficacy of existing treatment modalities for patients. The accurate identification of progressive glioma continues to present a significant challenge in the present time. Monitoring serum tumor markers is a highly advantageous approach for patients diagnosed with glioma due to its minimal invasiveness and safety. According to our findings, it has been observed that the utilization of serum-derived FAP obtained from patients diagnosed with glioma can serve as a viable method for disease detection. The monitoring of tumor response to treatment is of utmost importance, however, it presents significant challenges, particularly in glioma, which is recognized as the most aggressive variant of primary brain tumor. Neuroimaging predominantly serves as the primary means for assessing the diagnosis and therapeutic response of gliomas. Nevertheless, it is often observed that tumors subjected to surgical intervention and chemoradiotherapy tend to exhibit an increase in size during subsequent MRI examinations, thereby suggesting the occurrence of either progression or radiation necrosis (also known as pseudoprogression). The utilization of MR imaging or CT examinations is a commonly employed method, although it can occasionally lead to misleading outcomes and less than optimal accuracy.

The identification of serum tumor markers has recently gained prominence as a potential diagnostic tool for glioma [12]. The accuracy of image examination in assessing gliomas post-therapy is generally limited, whereas serum tumor markers have the potential to effectively compensate for this limitation, thereby alleviating the need for reoperation and reducing patient suffering [13, 14]. Tumor biomarkers present in circulating bodily fluids have the potential to serve as valuable tracers, facilitating the diagnosis process and enhancing the assessment of therapeutic outcomes in patients. Recently, there has been identification of circulating proteins released by tumor cells or cancer-associated cells as potential biomarkers for glioma. However, the current markers exhibit inadequate sensitivity and patient coverage, limiting their broad clinical applicability. [15] So far, little progress has been made in developing effective blood-based methods for tracking glioma. Though molecular and histological pathology based on tissue samples could provide accurate diagnosis and distinguish tumor markers for prognostic prediction, fluid-based tumor markers provide a minimally invasive approach for monitoring glioma without sampling tumors, despite its heterogeneity and evolution [16, 17]. In the current study, the potential diagnostic value of serum FAP detection as a marker was investigated in conjunction with tumor images. FAP is produced by human cancer-associated fibroblasts (CAFs) in tumors such as glioma. It, a transmembrane serine protease, is highly expressed in many tumors but completely absent in normal tissues [18, 19]. FAP has been identified as an independent biomarker associated with a poor prognosis in a growing number of cancers [20–23]. The presence of proangiogenic FAP in CAFs has been reported which is consistent with our findings [9, 12, 24].

CAFs cause the accumulation of FAP within tumors, consequently leading to an elevation in the level of FAP in the bloodstream. A group of researchers have identified a notable elevation in FAP levels among patients diagnosed with glioma [25]. According to the literature, the FAP expression in grade 2 gliomas is generally lower than that of patients with grade 3 and grade 4 gliomas, indicating that high-grade gliomas are associated with a high level of FAP expression [26]. To the best of our best knowledge, the utilization of the dynamic serum FAP test as a diagnostic tool for glioma detection has not been documented in existing literature. Due to the limited amount of research conducted on blood FAP for glioma trace, it is imperative to explore the potential of dynamic monitoring of tumor markers for clinical utilization. In comparison to an MRI examination, blood FAP test is less invasive, more accessible, inexpensive, and more convenient. In this regard, it would be extremely interesting for future studies to continuously track gliomas using serum FAP.

In order to explore the correlation between serum FAP expression and tumor characteristics, we conducted an analysis of serum FAP levels and their association with imaging observations. Our study demonstrates serum FAP levels in preoperative gliomas are significantly higher than those in postoperative patients, suggesting a positive correlation between serum FAP levels and tumor burden. The findings suggest that patients with tumor progression exhibit significantly elevated FAP levels compared to those without recurrent glioma, thereby highlighting the potential of blood tumor markers for glioma as a sensitive tool for early diagnosis. FAP expression in gliomas promotes tumor progression [24], though serum FAP levels vary. FAP-positive cells in immunohistochemical tests are spindle-shaped, fibroblast-like cells, which is consistent with our findings in gliomas [27]. Multiple studies conducted on stromal cells, specifically CAFs, within gliomas have revealed the presence of FAP expression in neoplastic glial cells [28]. In the majority of human solid cancers, the expression of FAP is observed in a selective manner among cancer-associated fibroblasts (CAFs) and pericytes, while tumor cells do not exhibit this expression [29]. The direct observation revealed the presence of prominent FAP staining in fibroblasts surrounding the tumor cells, while minimal or absent expression was observed in adjacent normal tissue. Due to its highly selective distribution in tumors, FAP served as a biomarker of reactive CAFs [29, 30]. Based on our research findings, there exists a positive correlation between the level of serum FAP and both the grade and molecular state of glioma. Multiple studies conducted on different types of cancers have revealed a strong correlation between elevated levels of FAP and the presence of cancer [31, 32]. The observed phenomenon was construed as a systemic reaction to the progression of the tumor [17, 33]. The occurrence of the homologous phenomenon was not documented in glioma.

Following surgical intervention and/or in conjunction with subsequent chemoradiotherapy, the evaluation of the disease predominantly relies on MRI, posing challenges in accurately discerning tumor progression from radiation necrosis within specific timeframes. While tissue biopsies are essential for precise diagnosis and molecular profiling, their limitations lie in their ability to solely capture a fixed moment in time, unable to consistently depict changes in the mutational spectrum, microenvironment, and heterogeneity evolution. The correlation between tumor volume and blood FAP levels suggests potential utility in guiding treatment strategy selection. The promotion of posttreatment glioma invasive growth by FAP suggests the existence of actively proliferating tumor cells [10]. Even if no obvious mass is visible on the MR image, blood FAP of glioma patient may serve as a tumor tracer.

Until now, the utilization of craniocerebral MRI scans has been suggested as a conventional diagnostic approach for post-treatment evaluation of gliomas. The utilization of MRI examination aids in the confirmation of the underlying cause responsible for the upregulation of FAP expression in gliomas. Investigating the origin of FAP detected in blood samples will contribute to a more comprehensive comprehension of its role as a protein biomarker. The dynamic serum FAP effectively addresses the limitations of MRI in differentiating between radiation necrosis and tumor progression. The integration of serial serum FAP test results with neuroimaging enhances the precision of early glioma recurrence detection, underscoring the potential of combining tumor markers with imaging as a viable approach in the clinical diagnosis of glioma. The early detection of glioma recurrence still remains challenging. In the present study, it was observed that serum FAP exhibited a progressive elevation in conjunction with the augmentation of tumor volume. This finding suggests that various cellular components implicated in glioma progression, including parenchymal cells, mesenchymal cells, and endothelial cells, might contribute to the synthesis of this protein.

In the present study, we conducted an analysis to determine the levels of FAP in the serum of patients diagnosed with glioma, and subsequently compared these levels with the assessments of tumor burden obtained through MRI imaging. The results of our study indicate a significant elevation in serum FAP levels as tumor progression occurs, suggesting that serum FAP has potential as a valuable tool for disease monitoring and as a marker for tumor progression. Additionally, our research reveals substantial variations in serum FAP levels among gliomas, with a notable elevation observed in a considerable proportion of high-grade gliomas compared to low-grade gliomas. Concurrently, a notable reduction in serum FAP level was observed in patients who did not experience tumor recurrence subsequent to successful treatment. The serum levels of FAP exhibited a significant increase in the presence of recurrent tumor, whereas the serum levels of FAP displayed fluctuations in accordance with the condition of the tumor. The utilization of longitudinal variations in serum FAP level in our analyses has led to the confirmation that serum FAP is a reliable indicator for evaluating the status of the disease. Another intriguing finding is that serum FAP level has a suggestive role in the molecular pathological subtypes of glioma. However, the association between serum FAP levels and MGMT promoter methylation status appears to be less definitive compared to other molecular statuses such as IDH and 1p/19q. We did not search for a direct link between MGMT status and blood FAP level in the literature. Our study found that serum FAP levels fluctuated in some cases while MRI assessments were stable. This phenomenon may be attributed to the constrained sensitivity of MRI in discerning minute masses within tumor dimensions, thereby requiring additional validation within a more extensive sample group.

Collectively, we conducted an investigation into the potential of serum-derived FAP obtained from patients with glioma to function as a biomarker for the disease. The results of our study unequivocally demonstrate that the dynamic detection of serum FAP serves as a straightforward approach to ascertain treatment response and evaluate tumor status. These findings suggest that serum FAP may be a potentially reliable biomarker for disease monitoring in the context of glioma, which is critical for the timely and accurate assessment of therapeutic effects. The incorporation of serum-derived FAP obtained from glioma patients, in conjunction with MRI evaluation, substantially enhances the precision of disease diagnosis.

## Data Availability

The data presented in this study are available on request from the corresponding author.
